# Effects of Cinnamon (*Cinnamomum zeylanicum*) Extract on Adipocyte Differentiation in 3T3-L1 Cells and Lipid Accumulation in Mice Fed a High-Fat Diet

**DOI:** 10.3390/nu15245110

**Published:** 2023-12-14

**Authors:** Joohee Oh, SongHee Ahn, Xiangqin Zhou, Yu Jin Lim, Sookyeong Hong, Hyun-Sook Kim

**Affiliations:** Department of Food and Nutrition, College of Human Ecology, Sookmyung Women’s University, Seoul 04310, Republic of Korea; joohee0131@sookmyung.ac.kr (J.O.);

**Keywords:** metabolic syndrome, cinnamon, anti-obesity, adipocytes, lipolysis, AMP-activated kinase (AMPK)

## Abstract

Flavonoids and phenolic acid are two of the rich polyphenols found in cinnamon (*Cinnamomum zeylanicum*). The effects of cinnamon extract on the inhibition of adipocyte differentiation in 3T3-L1 fibroblast cells and prohibitory lipid accumulation in male mice fed a high-fat diet were examined. Upon treating 3T3-L1 cells with cinnamon for 3 days, the cinnamon inhibited lipid accumulation and increased gene expression levels, such as those of adiponectin and leptin. In in vivo experiments, mice were randomized into four groups after a one-week acclimation period, as follows: normal diet, normal diet + 1% cinnamon extract, high-fat diet, and high-fat diet + 1% cinnamon extract. After 14 weeks of supplementation, we found that cinnamon extract increased the expression of lipolysis-related proteins, such as AMPK, p-ACC, and CPT-1, and reduced the expression of lipid-synthesis-related proteins, such as SREBP-1c and FAS, in liver tissue. Our results show that cinnamon extract may exhibit anti-obesity effects via the inhibition of lipid synthesis and adipogenesis and the induction of lipolysis in both 3T3-L1 fibroblast cells and mice fed a high-fat diet. Accordingly, cinnamon extract may have potential anti-obesity effects.

## 1. Introduction

Obesity is a lifestyle disorder caused by the excess fat that accumulates in tissues and the body due to unhealthy eating patterns, low physical activity levels, and unregulated sleep routines [1]. The major concern with obesity is dyslipidemia, defined as increasing triglyceride levels and low-density lipoprotein cholesterol (LDL-c) levels and decreasing high-density lipoprotein cholesterol (HDL-c) levels, which are important factors in hypertension and even cardiovascular diseases [2]. These pathologies are considered aspects of metabolic syndrome, which is prevalent worldwide [3]. They are also highly related to the dysregulation of lipid metabolism processes, such as lipid accumulation, lipolysis, adipogenesis, lipid synthesis, and fatty acid synthesis [4].

**Figure 1:**
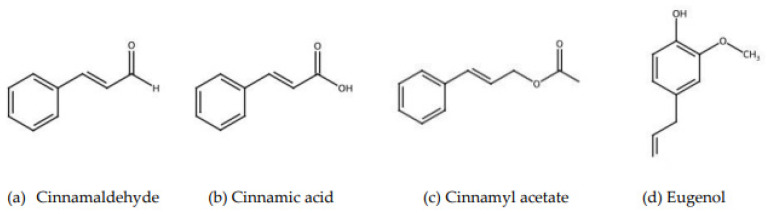
The main constituents of cinnamon [8,9,10].

Ceylon cinnamon (*Cinnamomum zeylanicum*) is native to Sri Lanka [5]. The area is famous for many widely utilized plant products, and it is also known for its biologically active substances [6]. Cinnamon has many health benefits, including anti-inflammatory properties, blood-glucose regulation, and anti-cardiovascular properties. It also lowers the risk of some cancers, as evidenced by numerous prior studies [7]. Ceylon cinnamon contains essential oils and other derivatives. In Figure 1, the main constituents of cinnamon, including cinnamaldehyde, cinnamic acid, cinnamyl acetate, and eugenol, are shown [8]. The chemical constituents of the different parts of cinnamon are shown in Table 1. In particular, the bark shows high amounts of cinnamaldehyde, ranging from 65.00 to 80.00% [9,10].

Cinnamon is very popular in most countries, not only as a cooking spice but also in traditional medicines and modern nutrition. Numerous research works have examined the antioxidant qualities of cinnamon, which has the ability to scavenge free radicals and inhibit lipid peroxidation and fatty acid oxidation [11,12]. Supplementing the diet with cinnamon has a variety of benefits, including anti-inflammatory, antioxidant, antidiabetic, antimicrobial, and anticancer effects. It also contains compounds that lower cholesterol and cardiovascular disease [13].

Lipid metabolism is a key source of lipid synthesis, lipid degradation, and the inhibition of lipid accumulation in liver steatosis [14]. White adipose tissue (WAT), where most excess fat in the body is stored, also plays a key role in secreting lipolysis or lipid synthesis hormones and inflammatory cytokines that regulate lipid metabolism [15]. Obesity is the excessive accumulation of fat in WAT, which can lead to dyslipidemia, adipocyte dysfunction, and cardiovascular disease [16]. A previous study demonstrated that the differentiation of adipocytes into mature adipocytes is highly related to obesity via fat production and accumulation [17]. The most popular choice for cells to study lipid accumulation, adipogenesis, and lipolysis are 3T3-L1 fibroblast cells, which can be used to study the differentiation of adipocytes and the associated anti-obesity effects [18]. In previous in vivo studies, several key markers of lipid metabolism have been identified. AMPK, the main marker of lipid metabolism, controls whole-body metabolism by downregulating the acetyl-CoA carboxylase (ACC) and carnitine palmitoyltransferase-1 (CPT-1) pathways, which regulate beta-oxidation [19]. ACC mainly catalyzes the synthesis of fatty acids by converting acetyl-CoA to malonyl-CoA [20]; at the same time, it blocks de novo lipogenesis and functions as an inhibitor of CPT-1 [21]. However, once ACC phosphorylates, it downregulates ACC activation [22] and restores CPT-1 activity [23]. Therefore, the AMPK, ACC, and CPT-1 pathways stimulate free fatty acid oxidation [24]. When the AMPK pathway activates, it prohibits the sterol regulatory element binding protein-1 (SREBP-1c), which regulates the genes for lipid production that are related to fatty acid synthase (FAS) [25]. Furthermore, another prior study showed that the liver’s production of lipids is reduced when the SREBP-1c and FAS pathways are downregulated [26]. Therefore, the pathways of AMPK, p-ACC, CPT-1, SREBP-1c, and FAS play crucial roles in lipid metabolism and prevent lipid accumulation in the organs and elsewhere in the body.

Many studies have reported on cinnamon’s antioxidant properties and its potential benefits for insulin disorders and diabetes; however, few studies have been carried out on the effect of cinnamon on obesity. An investigation of the effects of cinnamon (*Cinnamomum zeylanicum*) extract on adipocyte differentiation and lipid accumulation in 3T3-L1 fibroblast cells, its anti-obesity effect via the inhibition of adipogenesis and lipid synthesis metabolism, and its induction of lipolysis via the AMPK, p-ACC, CPT-1, SREBP-1c, and FAS pathways in male mice fed normal and high-fat diets is the aim of this study.

## 2. Materials and Methods

### 2.1. Materials

Pure Ceylon cinnamon (*C. zeylanicum*) powder (Wellsground Ceylon cinnamon; ingredients—pure Sri Lankan cinnamon powder; purity—100% cinnamon) from cinnamon bark that originated from Sri Lanka (Meewannapalana, Horana, Sri Lanka) was purchased from an Asian food market located in Ansan, Gyeonggi, Republic of Korea.

### 2.2. Preparation of Cinnamon Extract

The cinnamon powder (100 g) was dissolved in 1000 mL of 70% ethylene alcohol overnight at room temperature. This mixture was concentrated using a rotary vacuum evaporator (N-21NS; EYELA, Tokyo, Japan) at 40 °C after being centrifuged at 1902× *g* for 30 min and filtered through filter paper (5–8 µm, Qualitative, No. 2, Hyundai Micro) [27]. The concentrates were freeze-dried at −90 °C (Operon FDUT-8806, Gimpo, Gyeonggi, Republic of Korea) and stored at −20 °C prior to experimental use. Finally, a yield of 5.5% was sourced from the cinnamon extract.

### 2.3. Cell Culture

**Figure 2:**
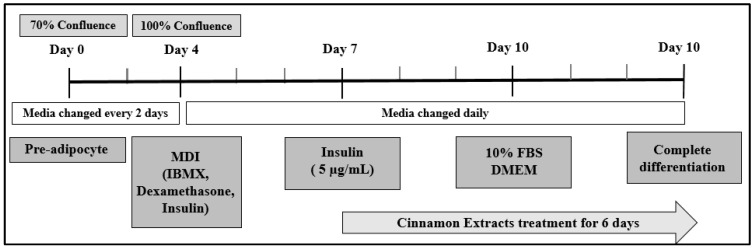
Cell culture design to determine the effect of cinnamon extract treatments on 3T3-L1 cells. Cells were treated with cinnamon extract after 3 days of differentiation and during adipogenesis for 6 days.

We purchased mouse 3T3-L1 fibroblasts from the American Type Culture Collection (ATCC, Manassas, VA, USA). They were grown under CO_2_ in an incubator (37 °C and 5% CO_2_) in Dulbecco’s modified Eagle’s medium (DMEM) supplemented with 10% bovine calf serum (BCS), along with a penicillin–streptomycin solution (100×) (GIBCO, Thermo Fisher Scientific, Inc.). For analysis, the cells were seeded and grown at a density of 1 × 10^5^ cells/cm^2^ in 6-well plates with DMEM and 10% fetal bovine serum (FBS; GIBCO, Thermo Fisher Scientific, Inc., Waltham, MA, USA) and antibiotics after 70% confluence (day 0) Figure 2. When the cells were 100% confluent, on day 4, the cells were differentiated using an MDI solution (5 µg/mL insulin, 0.5 mM 3-Isobutyl-1-methylxanthine, and 0.25 µM dexamethasone (Sigma-Aldrich, St Louis, MO, USA)) [28,29]. 

### 2.4. MTT Assay

Thiazolyl Blue Tetrazolium bromide solution (MTT; M2128) was used to determine the cell viability. In a 96-well plate, 3T3-L1 pre-adipocytes were seeded at a density of 1 × 10^4^ cells/cm^2^ with 10% BCS and 1% penicillin–streptomycin solution. After 12 h of incubation, the cells were treated with each concentration of cinnamon extract (1, 3, 5, 7, and 10 µg/mL). After 24 h of incubation, the MTT solution (5 mg/mL) was added. After a further 4 h, 200 µL of dimethyl sulfoxide (DMSO) was added to each well after the medium was suctioned. At 560 nm, absorbance was measured [30].

### 2.5. Oil Red O Staining

The lipid concentration of the cells was determined via Oil Red O staining. The 3T3-L1 pre-adipocytes were seeded in a 6-well plate at a density of 1 × 10^5^ cells/cm^2^. After 3T3-L1 cell differentiation, the cells were treated with each concentration of cinnamon extract (0, 1, 5, and 10 µg/mL). After 48 h of incubation, the cells were washed with PBS twice and fixed for 1 h in a 10% formalin solution. Following this, the cells were washed with 60% isopropanol, and the lipid droplets were stained for 30 min with an Oil Red O solution. The cells were washed with PBS and the stained fat droplets were visualized using an inverted microscope and then photographed. After using 100% isopropanol to dissolve the stained lipid droplets, the absorbance was determined to be 560 nm [31].

### 2.6. mRNA Expression Analysis

The 3T3-L1 pre-adipocytes were seeded in a 6-well plate at a density of 1 × 10^6^ cells/cm^2^. After 3T3-L1 cell differentiation, the cells were treated with each concentration of cinnamon extract (0, 1, 5, and 10 µg/mL). After 24 h of incubation, the mRNA expression was analyzed. RNA was extracted with RNAiso Plus (9108; TAKARA BIO Inc., Shiga, Japan) according to the following protocol. RNA purity was calculated using an absorbance ratio of 260/280 nm. For synthesizing the cDNA, the PrimeScript™ RT reagent kit (RR037A; TAKARA BIO Inc., Shiga, Japan) was used. The PCR products were mixed with TB Green Fast qPCR Mix (RR430A; TAKARA BIO Inc., Shiga, Japan) and analyzed using a LightCycler 96 (Roche Molecular Systems, Inc., Basel, Switzerland). For a relative quantitative analysis, each data point was normalized to *Gapdh*, and the fold changes were determined. The sequences are listed in Table 2.

### 2.7. Preparation of Diets

Raw Ceylon cinnamon (*C. zeylanicum*) powder, sourced from an Asian food market (Republic of Korea), was extracted via vacuum evaporation (N-21NS; EYELA, Tokyo, Japan) and freeze-dried before its use as cinnamon extract. The composition of this powder was determined by the Jeonnam Bioindustry Foundation Food Research Center before its use in this experiment, due to its use as a supplement in animal diets. A diet supplemented with cinnamon extract was created by modifying the AIN-93G diet (Research Diet, New Brunswick, NJ, USA) to a normal diet with 45% fat (Research Diet, New Brunswick, NJ, USA) to represent a high-fat diet. The percentage of cinnamon powder was determined as described in a previous study, which showed beneficial effects on type 2 diabetes mellitus and the conversion of animal doses into human-equivalent doses [32,33]. The normal diet group (ND) was fed with the normal diet (AIN-93G). The normal diet + 1% cinnamon powder (NC) was obtained by supplementing the normal diet + 1% cinnamon extract. The high-fat diet group (HF) was provided with a diet comprising 45% fat; the high-fat diet + 1% cinnamon powder (HC) was obtained by supplementing the high-fat diet + 1% cinnamon extract. The caloric values of the diet contents were changed but were equivalent for all diets and are presented in Table 3 and Table 4.

### 2.8. Animals and Housing Conditions

Forty male 6-week-old C57BL/6J mice (Saeron Bio, Gyeonggi-do, Republic of Korea) were housed in a 12-h light/dark cycle environment with 22 ± 1 °C temperature and 50–60% humidity. The mice were provided with access to water ad libitum. All experimental protocols were approved by the Institutional Animal Care and Use Committee (IACUC) of Sookmyung Women’s University regarding the care and use of laboratory animals (SMWU-IACUC-2006-006). 

### 2.9. Experimental Design of Animal Groups

Following a week of acclimation, the 7-week-old C57BL/6J mice were randomized into four groups (n = 10): ND, for mice fed a normal diet (AIN-93G); NC, for mice fed a normal diet (AIN-93G) supplemented with 1% cinnamon extract; HF, for mice fed a high-fat diet (45% fat); and HC, for mice fed a high-fat diet (45% fat) supplemented with 1% cinnamon extract. Each diet was administered daily for 14 weeks.

### 2.10. Measurement of Body Weight and Food Intake

Each week, the body weight of each mouse was measured. Food intake was measured every two days. The day before sacrifice, the final body weight was determined. The following equation was used to calculate the food efficiency ratio (FER):

FER (%) = total body weight gain (g/total food intake (g)) × 100

### 2.11. Blood and Tissue Collection

After the mouse was subjected to overnight fasting, the final body weight of each mouse was recorded. The mice were euthanized using CO_2_. Blood samples were collected in 5 mL SST tubes via cardiac puncture. After collection, the serum was centrifuged at 1902× *g* for 30 min (Combi-514R, Hanil Co. Ltd., Seoul, Republic of Korea). After the organs were removed, the weight of the brain, liver, lungs, kidneys, abdominal fat, and epididymal fat were recorded. All serum, organs, and fats were stored at −70 °C until analysis (DF8517; Ilshin Laboratory Co., Ltd., Seoul, Republic of Korea). Abdominal fat and epididymal fat were fixed in 10% formaldehyde for staining. The following formula was used to determine each organ’s coefficient:

Organ coefficient (g/100 g) = organ weight (g)/body weight (g) × 100

### 2.12. Lipid Profiles

Serum TG, TC, and HDL-C levels were measured using a kit of TG-S, T-CHO, and HDL-CHO, respectively (3I1570, 3I2020, and 3I2030; Asanpharm, Hwaseong, Republic of Korea).

Using the Friedewald equation [34], the levels of low-density lipoprotein and very-low-density lipoprotein cholesterol (LDL-C and VLDL-C) were determined, as follows:

LDL-C level (mg/dL) = TC level − (HDL-C level) + TG level/5

VLDL-C level (mg/dL) = TG level/5 (mg/dL)

### 2.13. Atherogenic Index (AI), Cardiac Risk Index (CRI) I, and II

The atherogenic index (AI), cardiac risk index I (CRI-I), and cardiac risk index II (CRI-II) were calculated using the following equations [35,36]:

AI = (TC level − HDL-C level)/HDL-C level

CRI-I = TC level/HDL-C level

CRI-II = LDL-C level/HDL-C level

### 2.14. Hepatic TG and TC Levels

The levels of hepatic TG and TC were measured using the Folch method to isolate lipids from the liver. Each liver sample was homogenized using a chloroform/methyl alcohol (2:1) reagent. The mixed samples were centrifuged for 5 min at 211× *g* at 20–25 °C. The supernatant was removed, and the remaining part was evaporated until the chloroform disappeared. The levels of hepatic TG and TC were examined using TG-S and T-CHO kits (3I2020, 3I1570; Asanpharm, Hwaseong, Republic of Korea), respectively.

### 2.15. Hormone-Sensitive Lipase in the Liver and WAT

Hormone-sensitive lipase (HSL) levels in the liver and WAT were determined using a mouse HSL ELISA kit (MBS720763, MyBioSource, San Diego, CA, USA).

### 2.16. Histological Analysis

The fat tissues of the abdominal and the epididymal samples were embedded in paraffin and fixed in a 10% neutral buffered formalin solution (Sigma-Aldrich Co., HT501128). The size of the adipose tissue was then measured by pathological examination using 5 µm-thick sections stained with hematoxylin and eosin (H&E). 

### 2.17. Western Blotting

To determine the effect of cinnamon extract on lipid metabolism by the liver tissue, 0.008 g ± 0.002 g of each sample was homogenized with 600 µL of Pro-prep protein extraction solution (17081, iNtRON, Biotechnology, Seongnam, Republic of Korea). The concentration of the protein was measured with a PRO-MEASURE ™ kit (21011, iNtRON Biotechnology, Seongnam, Republic of Korea). SDS-PAGE was used to load the sample and PVDF membrane (Merck Millipore, MA, USA) was used to transfer it, using an electrophoretic transfer system. Each membrane was blocked in a blocking buffer for 1 h and incubated overnight at 4 °C with each of the following diluted primary antibodies: AMPK antibody (1:500; 2532), phosphorylated AMPK antibody (1:1000; 2531), ACC antibody (1:1000; 3662), phosphorylated-ACC antibody (1:1000; 3661), FAS antibody (1:000; 3180), CPT-1 antibody (1:1000; 12252), SREBP1 antibody (1:1000; ab28481), and GAPDH (1:5000; 5174). Then, the membrane was washed every 10 min with 10× TBST three times. After that, the membrane was incubated for 2h with antirabbit IgG and horseradish peroxidase (HRP)-linked antibodies (secondary antibody, 1:3000; 7074). ECL Pico Plus (GBE-P200) was used for chemiluminescence detection and was analyzed via densitometric analysis (Amersham ImageQuant800, Thermo Fisher Scientific Inc., Waltham, MA, USA) [37].

### 2.18. Statistical Analysis

Statistical analysis was performed using Prism 10.0.2 (GraphPad Software Inc., La Jolla, CA, USA); the data are shown as means ± SD. The results for each group were analyzed using a one-way analysis of variance, followed by Tukey’s multiple comparison test, to determine the differences between groups. Significance was defined as follows: * *p*-value < 0.05, ** < 0.01, *** < 0.001, or **** < 0.0001.

## 3. Results

### 3.1. Effects of Cinnamon Extract on Cell Viability in Pre-Adipocytes

**Figure 3:**
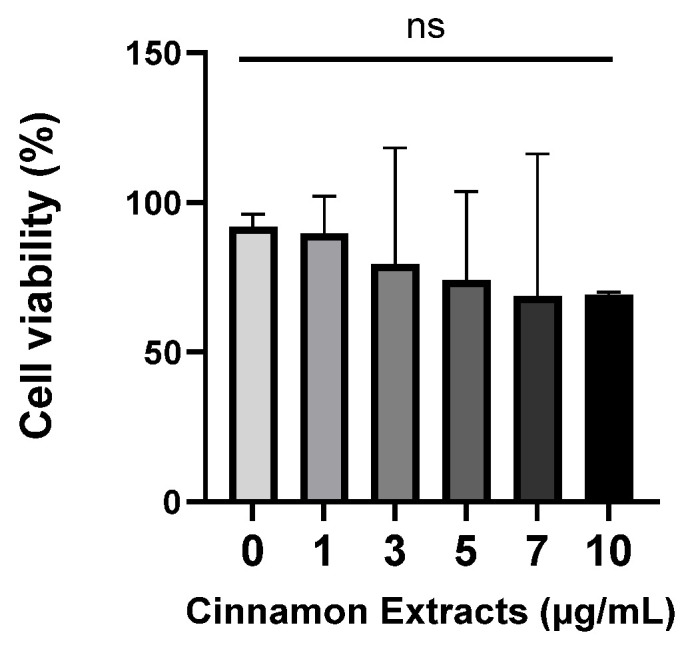
Effects of cinnamon extract supplementation (1, 3, 5, 7, 10 µg/mL) on 3T3-L1 pre-adipocytes. The data are expressed as means ± SD ns: Not significant.

The cell viability of 3T3-L1 pre-adipocytes was measured using an MTT assay. Cells were treated with different concentrations of cinnamon extract (1, 3, 5, 7, and 10 µg/mL). The cell viability showed no significant change at or below 10 µg/mL (Figure 3). Therefore, there is no toxicity at or below 10 µg/mL of cinnamon extract supplementation.

### 3.2. Effects of Cinnamon Extract on Lipid Concentration

**Figure 4:**
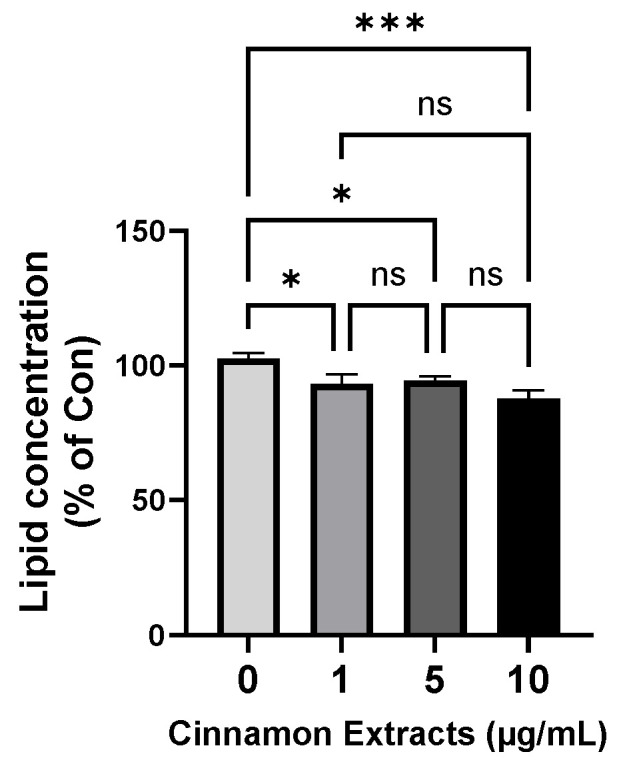
Effects of cinnamon extract supplementation (0, 1, 5, 10 µg/mL) on lipid concentration in 3T3-L1 cells, determined via Oil Red O staining. The data are expressed as means ± SD (*** *p* < 0.001, * *p* < 0.05). ns: Not significant.

The lipid concentration of the cinnamon extract in the 3T3-L1 cells was analyzed using Oil Red O staining (Figure 4) to determine the anti-lipid-accumulation effect of the cinnamon extract on adipocyte differentiation in 3T3-L1 cells. Pre-adipocytes were treated with an MDI solution for adipocyte differentiation and the lipid concentrations were analyzed. The results of treatment with different concentrations of cinnamon extract (0, 1, 5, and 10 µg/mL) suggest that 10 µg/mL of cinnamon extract supplementation significantly inhibited adipocyte differentiation, compared to 0 µg/mL (*p* < 0.05).

### 3.3. Effects of Cinnamon Extract on the mRNA Expression Levels of Genes Related to Lipid Metabolism: Antiadipogenic Effect, Lipolysis, and Lipid Synthesis in 3T3-L1 Cells

**Figure 5:**
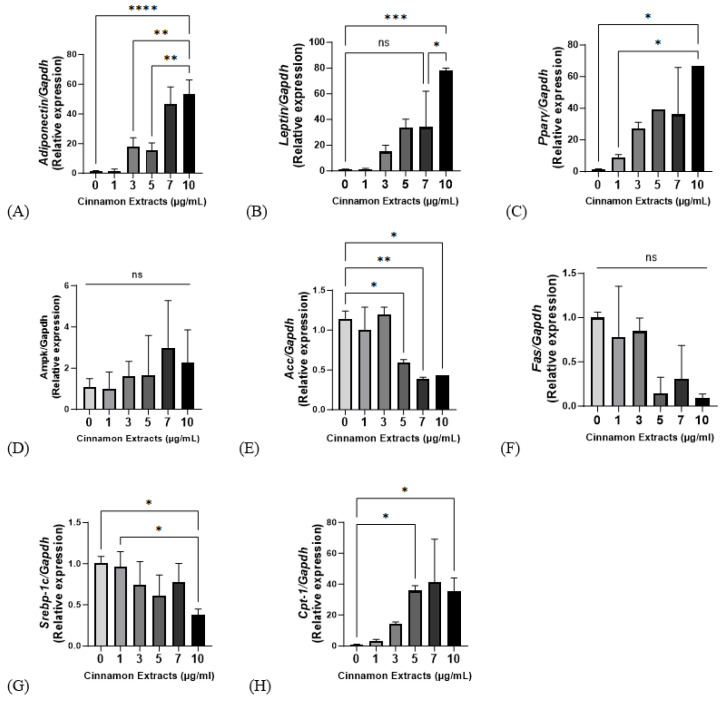
Effects of cinnamon extract (1, 3, 5, 7, 10 µg/mL) on mRNA gene expression, as related to lipid metabolism and adipogenic, lipolysis, and lipid synthesis in 3T3-L1 cells. mRNA expression levels of lipid metabolism, including *Adiponectin* (**A**), *Leptin* (**B**), *PPARγ* (**C**), and *Ampk* (**D**). mRNA expression levels of lipogenesis, including *Acc* (**E**), *Fas* (**F**), *Srebp-1c* (**G**), and lipolysis *Cpt-1* (**H**). The data are expressed as means ± SD (**** *p* < 0.0001, *** *p* < 0.001, ** *p* < 0.01, * *p* < 0.05). ns: Not significant.

Cinnamon extract (1, 3, 5, 7, and 10 µg/mL) treating groups provided with a diet supplemented with 10 µg/mL of cinnamon extract showed significantly higher gene expression levels in adiponectin, leptin, and PPARγ (*p* < 0.0001, *p* < 0.001, and *p* < 0.05, respectively) (Figure 5). *Ampk*, a regulator of lipid metabolism, has no significant changes but showed a higher trend at high doses. In the 10 µg/mL group, *ACC* showed significantly lower levels of gene expression than in the 0 µg/mL group, *FAS* showed a lower gene expression trend than in the 0 µg/mL group, and SREBP-1c showed a significantly lower expression level than in the 0 and 1 µg/mL groups. *CPT-1*, which regulates increased lipolysis, showed significantly higher levels in the 10 µg/mL group (*p* < 0.05).

### 3.4. Effects of Cinnamon Extract on Body Weight, Body Weight Gain, Food Intake, FER, and Organ Weight of Fat

**Figure 6:**
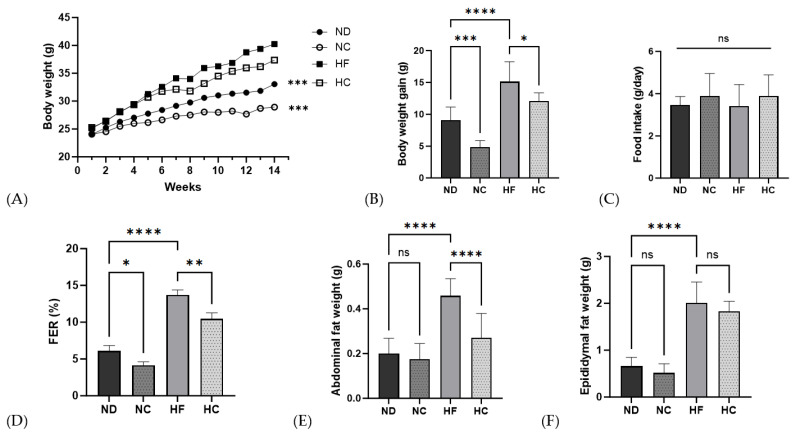
Effects of cinnamon extract on body weight, body weight gain, food intake, food efficiency ratio, and organ weight: body weight (**A**), weight gain (**B**), food intake (**C**), food efficiency ratio (**D**), organ weight, abdominal fat (**E**), and epididymal fat weight (**F**). FER (food efficiency ratio, %) = total body weight gain (g/total food intake (g)) × 100. Data are expressed as means ± SD (n = 10 for each group) (**** *p* < 0.0001, *** *p* < 0.001, ** *p* < 0.01, * *p* < 0.05). ns: Not significant.

The body weight, body weight gain, food intake, FER, and organ fat weight results are shown in Figure 6 and Table 5. There were no significant differences in initial body weights between the four groups. Throughout 14 weeks of dietary supplementation with cinnamon extract and each diet, the NC group showed the lowest weight among all groups (*p* < 0.001). The groups that received dietary supplementation with cinnamon extract, NC and HC, showed a significantly lower body weight-gain range than the ND and HF groups (*p* < 0.001 and *p* < 0.05, respectively). Food intake showed no significant difference between the groups throughout the 14-week supplementation period. The FERs of the cinnamon supplementation group showed a significantly lower rate than the not-supplied group or ND group (*p* < 0.05) and the HF group (*p* < 0.01). The HF group showed the highest abdominal fat weight (*p* < 0.0001) and no significant changes were seen in the ND and NC groups. The HF group showed the highest epididymal fat weight among all groups, but the difference was not significant. 

### 3.5. Effects of Cinnamon Extract on Serum Lipid Profiles

**Figure 7:**
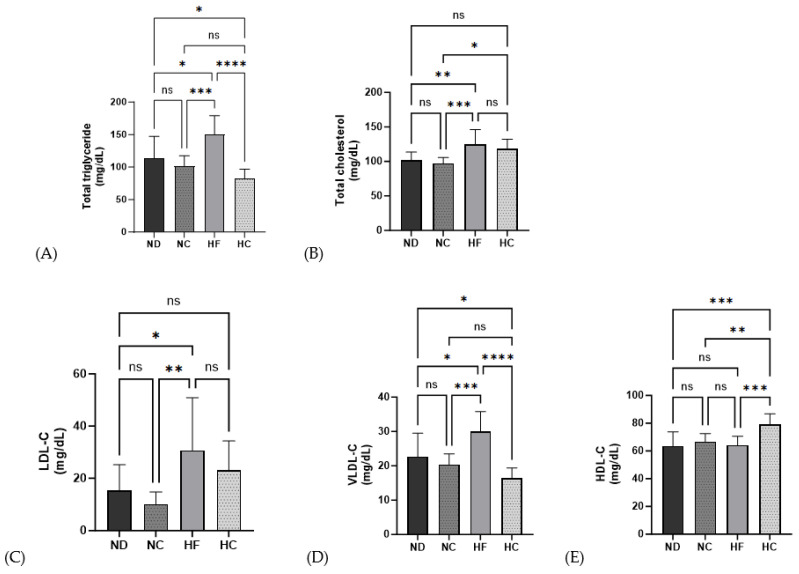
Effects of cinnamon extract on the serum biochemical lipid profiles of each group: total triglyceride (**A**), total cholesterol (**B**), LDL-C (**C**), VLDL-C (**D**), and HDL-C (**E**). Data are expressed as means ± SD (n = 10 for each group) (**** *p* < 0.0001, *** *p* < 0.001, ** *p* < 0.01, * *p* < 0.05). ns: Not significant.

As shown in Figure 7, the HF group demonstrated a significantly increasing level of lipid profiles for TG, TC, LDL-c, and VLDL-c compared to the ND group (*p* < 0.05, *p* < 0.01, *p* < 0.01, and *p* < 0.05, respectively), but no significant differences were found for HDL-c. The TG and VLDL-C levels in the HC group were significantly lower than those in the ND group and HF group (*p* < 0.05, *p* < 0.0001), while the TC and LDL-C levels showed no significant changes in the HC group compared to those in the ND group. The HDL-C levels in the HC group were significantly increased compared to those in the ND, NC, and HF groups (*p* < 0.001, *p* < 0.01, and *p* < 0.001, respectively).

### 3.6. Effects of Cinnamon Extract on the Atherogenic Index (AI) and Cardiac Risk Index (CRI-I, CRI-II)

**Figure 8:**
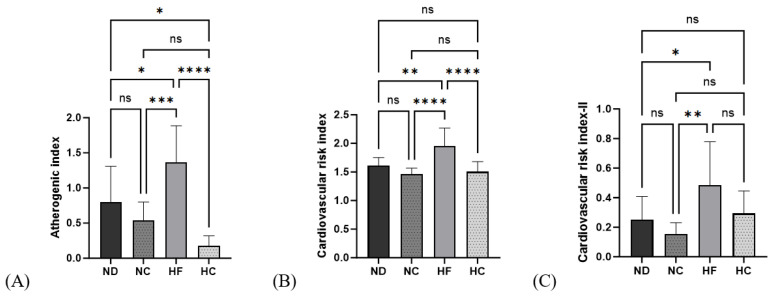
Effects of cinnamon extract on the atherogenic index (AI) and cardiac risk index (CRI-I, CRI-II): atherogenic index (**A**), cardiac risk index—I (**B**), and cardiac risk index—II (**C**). Data are expressed as means ± SD (n = 10 for each group) (**** *p* < 0.0001, *** *p* < 0.001, ** *p* < 0.01, * *p* < 0.05). ns: Not significant.

As seen from the data in Figure 8, AI, CRI I, and CRI II showed significantly higher values in the HF group than in the ND group (*p* < 0.05, *p* < 0.01, and *p* < 0.05, respectively). After 14 weeks of cinnamon extract supplementation, the AI in the HC group was significantly lower than that in the ND and HF groups (*p* < 0.05 and *p* < 0.0001, respectively). The cardiac risk index I in the HC group was significantly lower than that in the HF group (*p* < 0.0001) but showed no significant differences from the ND and NC groups. The cardiac risk index II in the HC group showed no significant differences from the ND and HF groups. 

### 3.7. Effects of Cinnamon Extract on Hepatic TG and TC Levels

**Figure 9:**
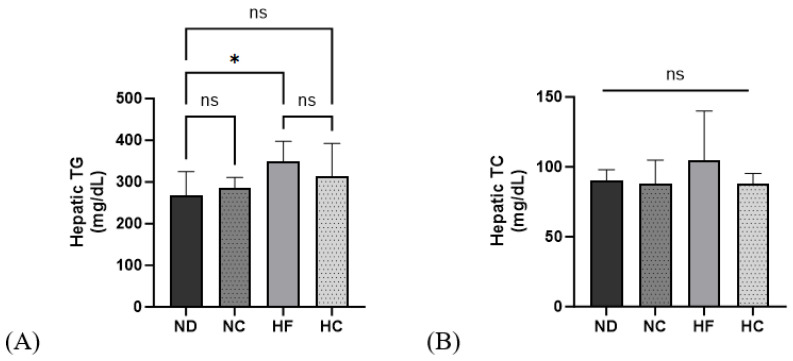
Effects of cinnamon extract on hepatic TG and TC levels: hepatic TG (**A**) and hepatic TC (**B**). Data are expressed as means ± SD (n = 10 for each group) (* *p* < 0.05). ns: Not significant.

In Figure 9, the determination of hepatic TG and TC levels are shown. The HF group showed the highest levels for both TG and TC; however, the difference was not significant. The HC group showed a decreasing trend compared to the HF group for both hepatic TG and TC.

### 3.8. Effects of Cinnamon Extract on HSL Activity in the Liver and WAT

**Figure 10:**
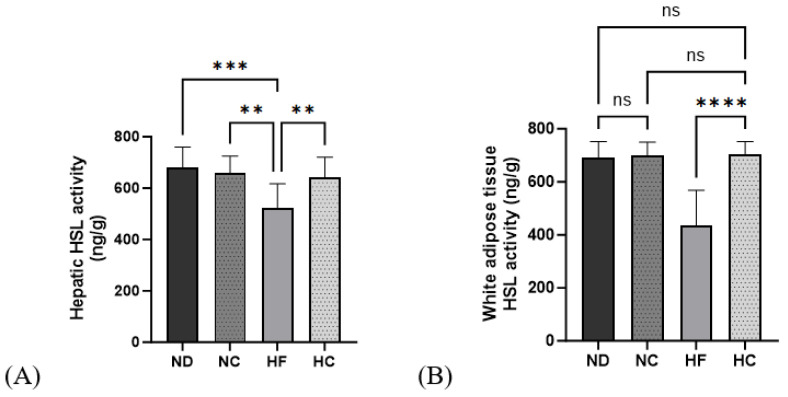
Effects of cinnamon extract on hormone-sensitive lipase activity in the liver and white adipose tissue: hepatic HSL activity (**A**) and white adipose tissue HSL activity (**B**). Data are expressed as means ± SD (n = 10 for each group) (**** *p* < 0.0001, *** *p* < 0.001, ** *p* < 0.01). ns: Not significant.

Figure 10 shows the results for hormone-sensitive lipases in the liver and WAT. The HSL activity in the HF group significantly decreased in the liver, and the HC group showed significantly higher levels than the HF group (*p* < 0.01). In the WAT, HSL activity in the HF group was the lowest among all groups; the HC group showed a significantly higher activity level than the HF group (*p* < 0.0001) and the ND, NC, and HC groups showed no significant differences in activity level. 

### 3.9. Effects of Cinnamon Extract on the Adipose Tissue Area (µm^2^) of Abdominal Adipose Tissue and Epididymal Adipose Tissue

**Figure 11:**
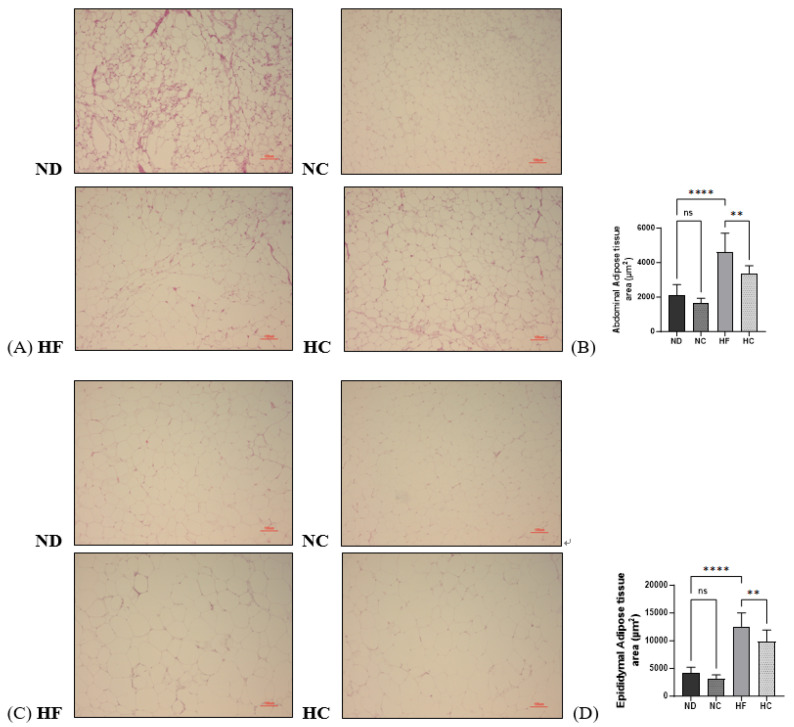
Effects of cinnamon extract on the adipose tissue area (µm^2^) of abdominal adipose tissue and epididymal adipose tissue: histological analysis of the abdominal fat (**A**), abdominal adipose tissue area (**B**), histological analysis of the epididymal fat (**C**), and epididymal adipose tissue area (**D**). Data are expressed as means ± SD (n = 10 for each group) (**** *p* < 0.0001, ** *p* < 0.01). ns: Not significant. The adipose tissue of H&E-stained samples is shown ×100.

A histological analysis via H&E staining of the abdominal adipose tissue and epididymal adipose tissue was performed; the samples were analyzed according to the adipose tissue area. The results are shown in Figure 11. As the data show, the sizes of both the abdominal adipose tissue and epididymal adipose tissue significantly increased in the HF group compared to the ND group (*p* < 0.0001) and decreased in the HC group compared to the HF group (*p* < 0.01). 

### 3.10. Effects of Cinnamon Extract on the Protein Expression Levels, as Related to Adipogenic, Lipolysis, and Lipid Synthesis Markers in the Liver

**Figure 12:**
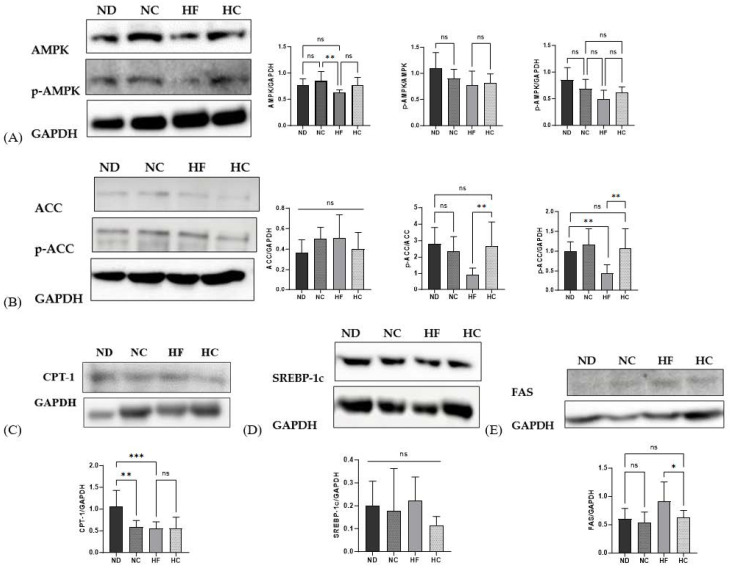
Effects of cinnamon extract on protein expression levels, as related to adipogenesis, lipolysis, and lipid synthesis in the liver: AMPK and p-AMPK (**A**), ACC and p-ACC (**B**), CPT-1 (**C**), SREBP-1c (**D**), and FAS (**E**). Data are expressed as means ± SD (n = 10 for each group) (*** *p* < 0.001, ** *p* < 0.01, * *p* < 0.05). ns: Not significant.

Figure 12 shows the protein expression levels of the adipogenic and lipolysis- and lipid synthesis-related markers in the liver. Western blotting was used to examine protein expression. The protein expression levels of AMPK, which primarily regulates lipid metabolism, showed no significant differences in all groups. Phosphorylated AMPK (p-AMPK) also showed similar expression levels among groups. ACC, which inhibits lipogenesis, showed increased levels in the HF group compared to the HC group, but this did not reach statistical significance. The levels of phosphorylated ACC (p-ACC), (which downregulates ACC to inhibit lipogenesis) in the HF group were the lowest among all groups (*p* < 0.01), while the HC group showed significantly higher expression levels for ACC than the HF group (*p* < 0.01). CPT-1 increases lipolysis and delivers fatty acids to the mitochondria for β-oxidation; the ND group showed the highest expression level among all groups (*p* < 0.01), but there were no significant differences between the cinnamon supplementation groups and the HF group. SREBP-1c, which controls lipid synthesis, showed the lowest levels in the HC group. In addition, the ND, NC, and HC groups exhibited no differences in protein expression. FAS, which regulates de novo lipogenesis, showed the highest level of expression for the HF group; the HC group showed significantly decreased levels compared to the HF group (*p* < 0.05), while the ND, NC, and HC groups showed no significant difference. 

## 4. Discussion

Obesity is a global public health disorder that increases the risk of chronic diseases due to metabolic syndrome, which occurs because of lipid accumulation in the tissues and fat of the body [1]. Cinnamon is rich in polyphenols such as flavonoids and inhibits lipid accumulation via its antioxidant effects [12,38]. Cinnamon is also rich in bioactive compounds that assist the metabolites and exert antioxidant and anti-obesity effects [39]. The present study was designed to demonstrate the effects of cinnamon extract on lipid accumulation, obesity, and lipid-related metabolic syndromes in 3T3-L1 cells and in male mice that were fed normal and high-fat diets.

For the in vitro study, the cell viability was measured and showed similar viability between the 0 and 10 µg/mL dosage range. Lipid accumulation is a marker of adipogenesis in pre-adipocytes, which produce and accumulate lipids in cells [1]. To demonstrate the amount of lipid accumulation, Oil Red O was analyzed, and the 10 µg/mL group showed the lowest lipid concentration. Examination of the anti-obesity effects of cinnamon extract, the relative expression levels were measured. Leptin and adiponectin play crucial roles in obesity-associated diseases, and the group that received the dose of 10 µg/mL showed significantly higher levels of adiponectin and leptin than the control group [40]. Because adiponectin is a homeostatic adipokine secreted by adipocytes that regulates lipid metabolism, a lack of adiponectin and leptin signaling can lead to cardiovascular problems [41,42,43]. 

The inhibitory regulation of lipid accumulation in 3T3-L1 cells, *Pparγ, Ampk, Acc, Cpt-1, Fas, and Srebp-1c* is the main role of lipid metabolism. *Pparγ*, regulating cellular differentiation, lipid metabolism, and glucose homeostasis [43], showed significantly higher levels in the 10 µg/mL group than the 0 µg/mL group but showed no differences in the 3–10 µg/mL dosage range. At a concentration of 10 µg/mL, the activation of *Ampk* and *Acc* and the inhibition of *Fas*, *Srebp-1c*, and *Cpt-1* may have potential lipid-accumulation effects [21]. 

In the in vivo study, regarding body weight, the HF group was the highest among all groups. Changes in body-weight gain were seen in the cinnamon-treated NC and HC groups, which showed lower body-weight gain than the non-cinnamon-treated ND and HF groups. In the present study, dietary supplementation with cinnamon extract led to weight loss, not only in the high-fat group but also in the ND group, even though the calorie intake was not significantly different between all groups. This shows that energy intake did not contribute to an increase in body weight; the FER (%) increased in the ND group compared to the NC group and in the HF group compared to the HC group, and the mice fed without cinnamon gained more weight than the mice provided with the same amount of feed but without supplementation [44]. The results also indicate that feeding the mice cinnamon extract lowered their abdominal fat weight and epididymal fat weight, which indicates the inhibition of lipid accumulation in the body.

Higher serum levels of TG, TC, LDL-C, and VLDL-C, and lower levels of HDL-C are associated with obesity and chronic disease [45]. In this study, the cinnamon-fed group showed decreased serum lipid levels, including TG, TC, LDL-C, and VLDL-C, and increased HDL-C levels. The cinnamon-extract-fed group had a lower risk index for AI, CRI, and CRI-II. High polyphenol content decreases the risk of dyslipidemia and cardiac diseases [46]. Fatty acid synthesis and lipid circulation via lipoprotein synthesis are all ongoing through the liver, which is a key hub for lipid metabolism [14]. Hepatic TG and TC levels are important indicators of NAFLD and were lower in the ND and HF groups than in the cinnamon-fed groups of NC and HC. Cinnamon supplementation decreased the hepatic lipid profile.

As demonstrated in a previous study [47], the overexpression of HSL could improve liver function for lipolysis in adipose tissue. In our study, HSL showed the lowest levels in the HF group compared to the ND, HC, and HC groups, which indicates improved liver function and lipolysis in the liver and WAT; the HF groups also showed a lack of HSL [47]. In this study, we found that excess lipids are stored in the abdominal and epididymal adipose tissues of patients with HF. Our study demonstrated that cinnamon supplementation reduces the size of adipose tissue.

According to a prior study, lipid metabolism is essential for metabolism in the liver, including lipid synthesis and lipid consumption, and for avoiding the accumulation of lipids in liver steatosis [18]. In this study, to determine the mechanism through which cinnamon extract inhibits lipid accumulation, adipogenesis, and the activation of lipolysis in HFD-induced mice, the protein expression of AMPK, p-AMPK, ACC, p-ACC, CPT-1, SREBP-1c, and FAS was examined. We found that AMPK and p-AMPK signaling was promoted to a greater extent in the ND, NC, and HC groups than in the HF group, promoting lipid catabolism and inhibiting lipogenesis by stimulating fatty acid oxidation [48]. AMPK inhibited ACC activity via phosphorylation [49]. Once the AMPK, p-ACC, and CPT-1 pathways are activated, fatty acid oxidation occurs [50]. The overexpression of SREBP-1c causes lipogenesis, which is correlated with the FAS genes that synthesize fatty acids [51]. In this study, the ACC in the HF group showed the highest protein expression levels among all groups, but the lowest activating expression levels were found for p-ACC. In addition, the cinnamon-supplemented group showed higher p-ACC expression than the non-supplemented group. The phosphorylation of ACC inhibits lipid accumulation by decreasing the concentration of malonyl-CoA [52] and also leads to an increase in CPT-1 [49]. A previous study demonstrated that malonyl-CoA limits lipogenesis because it is a precursor of lipid biosynthesis [53]. CPT-1 showed higher expression levels in the HC group than in the HF group, while no difference was observed between the ND, NC, and HC groups. It has been demonstrated that CPT-1 deficiency causes mitochondrial disorder or fatty acid oxidation [54]. SREBP-1c and FAS showed lower expression levels in the HC group than in the HF group, demonstrating that glucose inhibits lipid synthesis in the liver [55]. 

Overall, our study provides evidence that cinnamon extract supplementation promotes lipolysis and inhibits lipogenesis and lipid accumulation. A previous study demonstrated the beneficial effects of cinnamon on type 2 diabetes [56], while this study demonstrated that cinnamon modulates AMPK, p-ACC, and CPT-1, downregulating or inhibiting the ACC, SREBP-1c, and FAS pathways, promoting lipolysis and fatty acid oxidation and inhibiting adipogenesis and lipogenesis [21]. All these are actions that have beneficial effects on dyslipidemia, metabolic syndrome, and the prevention or treatment of obesity.

## 5. Conclusions

In this study, we showed that cinnamon supplementation inhibited lipid accumulation and lowered the gene expression of adipogenesis but increased lipolysis in 3T3-L1 cells. Moreover, its anti-dyslipidemia effects were confirmed via a decrease in VLDL-c and an increase in HDL-c, thereby lowering fat accumulation in the body and fatty tissues and decreasing the risk of cardiovascular diseases. Cinnamon supplementation induced lipolysis by downregulating lipogenesis and upregulating lipolysis in the livers of obese mice. These findings demonstrate that cinnamon extract has beneficial effects on obesity. 

However, this study has some limitations. Since cinnamon powder is widely used as a spice and not as a primary food component, there is a need to promote cinnamon powder intake in the form of tablets or tea. It is necessary to investigate the impacts of such alternate forms of consumption of cinnamon on human health. Although further clinical studies are needed to understand the effects of cinnamon, our results provide meaningful data for anti-obesity and metabolic disorder treatments.

## Figures and Tables

**Table 1:** Chemical constituents of the different parts of cinnamon [8].

Part of Cinnamon	Compound	Percentage
Leaves	Cinnamaldehyde	1.00–5.00
	Eugenol	70.00–95.00
Bark	Cinnamaldehyde	65.00–80.00
	Eugenol	5.00–10.00
Root bark	Camphor	60.00
Fruit	Trans-Cinnamyl acetate	42.00–54.00
	Caryophyllene	9.00–14.00

**Table 2:** Primers designed for quantitative real-time PCR.

Target Gene	Primer Sequence
*Leptin*	Forward: 5′-GGA CTT CAT TCC TGG GCT TCA-3′ Reverse: 5′-TGC AGC ACA TTT TGG GAA GG-3′
*Adiponectin*	Forward: 5′-ACT ACC TGC TAC ATG GCC ACA-3′ Reverse: 5′-AGC AGA TGT GTC CAG ATG TTG-3′
*Pparγ*	Forward: 5′-CAC CAA CTT CGG AAT CAG CTC-3′ Reverse: 5′-CAA CCA TTG GGT CAG CTC TTG-3′
*Ampk*	Forward: 5′-ACC TGA GAA CGT CCT GCT TG-3′ Reverse: 5′-GGC CTG CGT ACA ATC TTC CT-3′
*Acc*	Forward: 5′-ATG GGC GGA ATG GTC TCT TT-3′ Reverse: 5′-TGG GGA CCT TGT CTT CAT CA-3′
*Fas*	Forward: 5′-TGC ACC CTG ACC CAG AAT AC-3′ Reverse: 5′-CGG CTC AAG GGT TCC ATG TT-3′
*Cpt-1*	Forward: 5′-GCC ACT GAT GAA GGA GGG AG-3′ Reverse: 5′-AAT TTG TGG CCC ACC AGG AT-3′
*Srebp-1c*	Forward: 5′-GCG CTA CCG GTC TTC TAT CA-3′ Reverse: 5′-TGC TGC CAA AAG ACA AGG G-3′
*Gapdh*	Forward:5′-CCG TGT TCC TAC CCC CAA TG-3′ Reverse: 5′-GTT GCT GTT GAA GTC GCA GG-3′

Abbreviations: *Pparγ*, peroxisome proliferator-activated receptor gamma; *Ampk*, AMP-activated kinase; *Acc*, acetyl-CoA carboxylase; *Fas*, fatty acid synthase; *Cpt-1*, carnitine palmitoyltransferase-1; *Srebp-1c*, sterol regulatory element binding protein 1; *Gapdh*, glyceraldehyde-3-phosphate dehydrogenase.

**Table 3:** Nutritional components of cinnamon extract.

	Cinnamon Extract
Calories (kcal/100 g)	368.9
Sodium (g/100 g)	0.1
Carbohydrate (g/100 g)	90.7
Sugars (g/100 g)	8.4
Crude fat (g/100 g)	0.1
Saturated fat (g/100 g)	0.0
Crude protein (g/100 g)	1.3
Moisture (g/100 g)	2.1
Crude ash (g/100 g)	5.8

The nutritional composition of the cinnamon extract was measured by the Jeonnam Bioindustry Foundation Food Research Center (Jeollanam-do, Republic of Korea); all values are presented as means.

**Table 4:** Composition of experimental diets.

Group	ND	NC	HF	HC
**Macronutrient composition (%)**				
Protein, % of energy	21.0	20.9	20.0	20.1
Fat, % of energy	16.0	15.9	45.0	44.6
Carbohydrate, % of energy	63.0	63.2	35.0	35.3
Energy density, kcal/kg	3960	3996.9	4700	4689.9
**Ingredients (g)**				
**Protein**				
Casein, lactic, 30 mesh	200.0	198.0	233.1	230.7
Cystine, L	3.0	3.0	3.5	3.5
Protein from cinnamon extract	-	0.13	-	0.13
**Carbohydrate**				
Starch, corn	397.5	393.5	84.8	84.0
Lodex 10	132.0	130.7	116.5	115.4
Sucrose, finely granulated	100.0	99.0	206.0	204.0
Carbohydrate from cinnamon extract	-	9.9	-	9.9
**Fiber**				
Solka Floc, FCC200	50.0	49.5	58.3	57.7
**Fat**				
Soybean oil, USP	70.0	69.3	29.1	28.8
Lard	-	-	206.8	204.8
Fat from cinnamon extract	-	0.01	-	0.01
**Mineral**				
S10022G	35.0	34.7	-	-
S10026B	-	-	58.3	57.7
**Vitamin**				
V10037	10.0	9.9	-	-
V10001C	-	-	1.2	1.2
Choline bitartrate	2.5	2.5	2.3	2.3
**Dye**				
Red FD&C #40	-	-	0.06	0.06
Alum. lake 35-42%				
**Total (g)**	**1000.0**	**1000.14**	**999.96**	**1000.14**

AIN-93G was used as the control diet for normal diet groups. A rodent diet with 45 kcal% fat was used for the high-fat diet groups. ND, normal diet group, NC: normal diet + 1% cinnamon extract group; HF, high-fat diet; HC, high-fat diet + 1% cinnamon extract group.

**Table 5:** Test results for body weight, body weight gain, food intake, the food efficiency ratio, and organ weight. Data are expressed as means ± SD (n = 10 for each group) (**** *p* < 0.0001, *** *p* < 0.001, ** *p* < 0.01, * *p* < 0.05). ns: Not significant.

Group	ND	NC	HF	HC
Initial body weight (g)	24.00 ± 1.68	24.07 ± 1.24	25.11 ± 1.58	25.26 ± 1.07
Final body weight (g)	33.06 ± 2.55 ***	28.93 ± 1.40 ***	40.23 ± 4.19	37.37 ± 2.14
Body weight gain (g)	9.06 ± 2.09 ****	4.86 ± 1.02 ***	15.12 ± 3.15	12.11 ± 1.25 *
Food intake (g/day)	3.46 ± 0.41	3.88 ± 1.07	3.41 ± 1.02	3.89 ± 0.99
FER (%)	6.11 ± 0.73 ****	4.15 ± 0.45 *	13.72 ± 0.67	10.46 ± 0.81 **
Abdominal fat (g)	0.20 ± 0.07 ****	0.18 ± 0.07 ^ns^	0.46 ± 0.08	0.27 ± 0.11 ****
Epididymal fat (g)	0.66 ± 0.19 ****	0.52 ± 0.19	2.01 ± 0.44	1.83 ± 0.21

## Data Availability

The data presented in this study are available on request from the corresponding author. The data are not publicly available due to privacy.
